# 

**Published:** 1894-08-25

**Authors:** 


### CONTENTS.

**Operative Surgeons.—The Great Untaught** - 421

**Around the Hospitals** - 422

**Modern Medico-Psychology and Psychiatry.** By T. S. Clouston, M.D.—Condition of Mental Exaltation and Excitement - 423

**Health and Holiness** - 424

**Annotations.**—Work for the Duke of York—The Holiday Season—The Dangerous Bicycle - 425

**Medical Progress and Hospital Clinics:**

The Treatment of Fractures by Some of the Simpler Methods - 427

Progress in Medicine.—Diseases of the Vascular System - 428

Progress in Surgery.—Bone, Joint, and Orthopædic Surgery - 430

**Notes and News** - 426

**The British Medical Association at Bristol.**—The Annual Museum - 431

**New Appliances and Things Medical** - 432

**The Institutional Workshop:**

Practical Departments.—Fry's Cocoas and Chocolates - 433

The Story of the Insane from Year to Year - 434

Hospitals in India - 435

Ethics and Administration - 435

**The Editor's Letter Box** - 436

**Scraps and Gleanings** - 436

**The Book World of Medicine and Science** - 437

### EXTRA SUPPLEMENT.—THE NURSING MIRROR.

**News from the Nursing World.**—Our Christmas Competitions—Prudent Precautions—A Cottage Hospital Appreciated—Worthy of Imitation—Through Improper Mediums—Recommendations "Emphasized"—Time Off Duty—A Good Woman—Seaside Experiences—Short Items - ccxi

**On General Nursing.—XXVI.** Obstruction of the Bowels ccxiii

**Structure and Care of the Teeth.—III.** Deterioration - ccxiv

**Worthing** - ccxiv

**Old Time Nurses.**—III. Impediments to Saving - ccxv

**Appointments** - ccxv

**Waiting—Progress at Northampton** - ccxvi

**Miss Kate Marsden—For Reading to the Sick** - ccxvi

**Character Sketches from a Lunatic Asylum.**—The Patient Who was Haunted by Spirits - ccxvii

**Holidays in Japan.**—II. The Journey - ccxvii

**A Case for Assistance—Notes and Queries** - ccxviii

**The Muses' Looking Glass** - ccxix

**Everybody's Opinion** - ccxx

